# The Diseases of the Pancreas

**Published:** 1844-10

**Authors:** 


### Art. X.

*Die Krankheiten der Bauchspeicheldrüse, etc.* Von D. H. Claessen. —*Köln*, 1842.

*The Diseases of the Pancreas.* By Dr. H. Claessen.—*Cologne*, 1842. 8vo, pp. 368.

Although several valuable contributions to the pathology of the pancreas have been made from time to time in this country by Abercrombie, Bright, Sandwith, Annesley, and others, nothing like a general treatise on the diseases of this organ has been attempted in the English language, with the exception of a short paper by Dr. Bigsby, in the Edinburgh Medical and Surgical Journal for 1835. On the continent several systematic treatises on diseases of the pancreas have been published, the first in Germany in 1812, by Harless,* and more recently by Bécourt and Mondière in France; to these we have now to add the treatise of Dr. Claessen, the most complete which has yet appeared on this confessedly obscure part of pathology.

As the object of our present notice is rather to give a condensed view of the contents of Dr. Claessen's work than to criticise it, we shall content ourselves with observing that it exhibits both the merits and defects due to its German parentage; while, on the one hand, it presents us with a laborious collection and examination of materials from all sources; on the other, it is certainly obnoxious to the charge of prolixity, which we are not unfrequently obliged to bring against the productions of our learned brethren beyond the Rhine.

The introductory part of Dr. Claessen's work is occupied by a short historical sketch of the opinions and observations of the earlier anatomists respecting the pancreas, and with an account of its anatomy and physiology, at least as far as the latter is at present understood. On these points we discover nothing possessing sufficient novelty to detain us. In regard to the opinions so prevalent amongst foreign authors, viz. that the pancreas is strictly analogous to the true salivary glands—that like these it is liable to be morbidly excited by the action of mercury, and when so excited to give rise to frequent watery colourless or greenish stools—and, lastly, that morbid actions are frequently transferred from the one to the other of these glands; our author maintains that many of the facts on which these opinions are founded have been misunderstood, and that the rest are by no means sufficient to justify the very decided manner in which they have been received as true. We are inclined with our author to think that the data on which these doctrines are founded, especially the two latter, are not sufficient to justify their reception without further question, a point on which it may be the more necessary to insist, as they have been lately introduced, with approbation, to pathologists in this country by Dr. Battersby.†

The work before us is arranged in two divisions: The first contains considerations on diseases of the pancreas in general, under the respective heads of occurrence, duration, symptomatology, complications, di-

* Abhandlungen der phys. mediz. Soc. zu Erlangen.

† See a paper on Scirrhus of the Pancreas, in a recent number of the Dublin Journal.

agnosis, prognosis, and cure. The second division contains a special exposition of diseases of the pancreas, whether functional, inflammatory, attended with morbid change of structure, or arising from morbid growths.

1. *Statistics and etiology.* Dr. Claessen doubts the correctness of the general opinion that affections of the pancreas are very rare, and thinks it has arisen rather from the difficulty of their diagnosis than their non-existence. In corroboration of this opinion, he states that though he had without difficulty collected reports of 300 cases in which disease of the pancreas was discovered after death, he had found only twenty instances in which the physician had ventured from symptoms alone to announce the existence of diseases of this organ, and that in very few of these the diagnosis appeared to have been correct. The size of the gland, and the important function with which it is connected, would also lead us to expect it to be not unfrequently diseased. The evidence which can be offered in favour of hereditary influence is not great, but rather in its favour. Of 322 cases collected by Dr. Claessen, 193 were in men, 129 in women.

In 262 cases in which the age is stated, the disease occurred,

| | | |
|---|---|---|
| In newborn infants | 5 | times. |
| In the first year | 2 | ,, |
| From 1 to 10 years | 20 | ,, |
| 10 to 25 ,, | 41 | ,, |
| 25 to 60 ,, | 156 | ,, |
| After 60 | 38 | ,, |

In many of the narratives, excess in food and drink, especially the latter, is stated to have been the exciting cause of the disease. The action of mercury, the abuse of tobacco, and the long-continued use of cinchona bark, have been supposed to give rise to disease of the pancreas.

In several cases, sudden stoppage of the menstrual discharge was speedily followed by affection of this gland.

Scrofula seems to be a frequent cause of these diseases in children. Metastasis from the salivary glands and from the testicles is thought by some authors to give rise to pancreatic diseases.

Gout and rheumatism have each been accused as occasionally producing similar effects.

A frequent cause of these diseases is supposed to exist in extension of morbid action from neighbouring organs, as the duodenum, the under surface of the liver, &c. to the pancreas. This cause Dr. Claessen thinks much overrated, and considers that the evidence goes rather to show that there is an indisposition on the part of the pancreas to take on the diseases of neighbouring parts, in evidence of which he cites several instances in which the pancreas was found healthy, though surrounded and pressed on by malignant and other diseases of neighbouring parts.

2. *Symptomatology and diagnosis.*

"In a certain number of cases (51) the pancreas was found more or less altered in structure, without any symptom having occurred during life to lead one to expect this. In some instances the patient had died from disease of a distant part, as of the viscera of the head or chest; in some, from well-marked acute abdominal disease; whilst in others, the organs adjoining the pancreas had been chiefly affected, and had given rise to a set of symptoms none of which seemed particularly attributable to the pancreas. From this latter class of cases the conclusion has been attempted to be drawn, that in complications of pancreatic diseases, with diseases of the neighbouring organs, the former are prevented from manifesting themselves, and are supplanted and concealed by the symptoms of the latter affection."

Our author combats this as a general proposition, and states that in many of these cases the change of structure in the pancreas consisted only in some trifling alteration of size or consistence, which scarcely merited to be considered as morbid. He, however, admits that a suspension of the function of this gland may take place without any apparent disorder of the operation of digestion, and this even when the suspension is caused by the invasion of the gland itself by abnormal formation. This is, however, by no means peculiar to the pancreas.

In the majority of cases, however, organic disease of the pancreas does not exist without giving rise to marked disturbance, first of the digestive functions, and by reflected action in more distant parts.

In order to arrive at satisfactory conclusions respecting the symptoms to which diseases of the pancreas give rise, it was not only necessary to select cases in which the diagnosis had been confirmed by a necropsy, but also to limit the choice to those in which either the pancreas was the only part diseased, or the affections of other organs were too trifling to give rise to doubt whether these might not have been the cause of the symptoms. Seventy such cases were selected by our author.

*Local abnormal sensations.* These were present in 63 cases, but their character varied as well as their situation. In 28 cases the pain was referred to the region of the stomach, and consisted of either a mere feeling of discomfort or of pressure, stretching or distension in that part; in some cases the feeling was more acute, like burning or cutting, sometimes existing spontaneously, at others excited by pressure. In 12 cases the pain was above the navel; in 15 cases behind the stomach, near the spine, and extending to each side, so as to resemble pain in the kidneys; in 9 cases the pain was more changeable in situation.

These differences did not arise from the presence or absence of disease of the stomach or other organs; and the presence of pain in so large a proportion of these cases shows that even where disease exists at the same time in other parts, the pain may yet fairly be attributed to the pancreatic affection and not to the accompanying disease. "The nature of the pain is very variable, and may be either a dull pressure at the epigastrium; heat and anguish in the precordia, causing incessant restlessness; tension, as of a weight suspended from the stomach; the sensation of a heavy body sinking in as the patient turns on the side," &c.; sometimes the pain is more acute, as of a dog gnawing the vitals, and may even be so agonizing, as almost to cause madness and a desire to tear the flesh.

The pain in duration and severity was altogether independent of external influences, though in several cases it was increased by pressure with the hand or by a loaded state of the stomach or arch of the colon. Occasionally, the position of the body influenced the pain. In general it was constant, in some cases fearfully so, enduring day and night for years. It was generally one of the earliest symptoms. Pain was attended with vomiting in 35 cases; with constipation in 24; with watery evacuations from the mouth in 16; with thirst in 12; with diarrhea in 7; and existed alone in 5.

The great depth at which the pancreas is placed makes it often impossible to distinguish the presence of enlargement of the organ, even when this is considerable; especially if the patient remains tolerably fat, or if dropsy is present. A position on the hands and knees—no meal having been taken for some time—and the bowels having been previously emptied by injections, as Annesley advises, will facilitate the examination. The position of the swelling varies much in different cases: generally it will be found situated above the navel, but, where the tumour is large, may extend into the right or left hypochondrium, or right or left flank. In several instances pulsation, synchronous with the heart's action, was felt.*

Thirty cases presented, in a greater or less degree, a *watery discharge from the mouth*, in seven cases with the characters of well-marked pyrosis. In one of these no other symptom of affection of the stomach was present throughout the disease; but in the others the character of the affection gradually changed into vomiting of watery fluid and food, or continued along with these. Generally the pain terminates like a cramp, with a more or less abundant discharge of thin and watery fluid, or of tough, ropy, spittle-like or slimy fluid—tasteless or sourish, sourish and bitter, or decidedly acid—colourless or brown, tinged with blood, yellowish, green, or of a dark greenish hue. Authors have differed in opinion respecting the source of this secretion. Some, as Wichman and Rahn, attribute it to the salivary glands which they suppose to take on a vicarious action on the cessation of the pancreatic secretion. Others conceive it to have its source in the pancreas, and have accordingly called it *sialorrhœa* of the pancreas. Our author objects to both these views. Against the former he adduces the fact, that though at first, in some few cases, the fluid filled the mouth without the patient having been aware of its ascent through the œsophagus, in the further progress of these, fluid of the same kind was distinctly thrown from the stomach by vomiting. In half of the whole number the evacuation was from the first accompanied by vomiting, whilst in five others the patient was sensible of its ascent through the œsophagus. In none of the cases, moreover, was any other symptom of excitement of the salivary glands present.

"In only seven cases do the writers speak of the symptom decidedly as a flow of saliva. In three of these it is simply called so, without any mention of a careful examination of its nature having been made; in two others *vomiting* accompanied the secretion, which may lead us to suspect a mistake on the part of the observer. In one case an involuntary flow of saliva was thought to have set in shortly before death; lastly, in one case, which was attended with mental disorder, the patient spat a large quantity of saliva, night and day. In three other cases in which this symptom is mentioned, we are furnished with no means of judging as to the correctness of the designation."

These cases, our author thinks, give no sufficient support to the

* In a case related by Dr. Battersby in the Dublin Med. Journal, vol. xx, p. 219, the pulsation was so marked, attended with bruit de soufflet, as to lead to the opinion that the case was one of abdominal aneurism. After death, a cyst was found in the pancreatic tumour.

notion of vicarious action of the salivary glands in pancreatic disease, founded on the supposed sympathy between these organs. In the great majority of instances it was evident that the fluid ascended the œsophagus; and we have therefore to choose between the stomach and the pancreas as the source of the secretion. Harless, Heischmann, Schmackpfeffer, and others have supposed the pancreas to be the source of the secretion, having been led to adopt this view from happening to meet only with cases of partial hardening of the pancreas. But numerous cases are to be found detailed in which this discharge of fluid was present in a marked degree, in which the structure of the pancreas was so entirely destroyed as to render secretion out of the question. To this we may add, that vomiting of a watery or slimy fluid is a not unfrequent symptom of disease of the stomach itself, of which some remarkable examples are detailed by Andral in his Clinique Médicale. For these and other reasons our author comes to the conclusion, that the stomach is the source of the rejected fluid. This fluid is often thrown off in immense quantities. Frank saw a case in which six pounds of it were brought up in a day; and Trumpes relates one in which from two to three pounds were evacuated every two hours.

In the 30 cases in which this symptom existed, we find it attended with vomiting in 17; pain in 16; costiveness in 11; diarrhea, attended with costiveness, in 4; diarrhea in 3; loss of appetite in 3; variable appetite in 2.

It will be observed how frequently these watery evacuations are accompanied by pain and costiveness. "The latter," observes our author, "will be shown to be especially connected with inflammation of the pancreas, and this also holds true as regards pain; from which it will be apparent that the increased secretion of the stomach is in most cases excited by inflammatory action in the pancreas."

*Thirst and hunger.* There are other symptoms which, from their nature, imply disorder of the functions of the stomach, but which are so constantly present in affections of the pancreas as to be necessarily reckoned amongst the essential symptoms of these diseases; and it is evident that, in studying them, only those observations should be selected in which the stomach was found, after death, to exhibit either no sensible change, or only changes of such an unimportant character as not to require to be taken into account. The first to be considered is the disturbances of those sensibilities of the stomach which exhibit themselves as thirst and hunger. Of 28 cases in which the state of the appetite is noticed, we find 19 in which it was either lost or remarkably diminished. In 6 cases the appetite was natural or increased. In 3 cases want of appetite, alternated with natural desire for food. This is never the only symptom present. Those cases in which the appetite was natural were attended with fewest symptoms, whilst of the 19 cases in which a marked diminution or loss of appetite occurred, the following was the proportion in which this was joined with other symptoms: pain in 17 cases, vomiting in 11, costiveness in 7, thirst in 5, watery evacuations from the mouth in 4, diarrhea in 3. Thirst was present only in 12 cases; and in these it appeared rather to accompany a feverish state of the system than as a special symptom of the pancreatic affection.

The state of the tongue was noticed in very few of the above cases, and no particular connexion could be traced between loss of appetite or thirst and a loaded or clean and moist state of the tongue.

"The tongue is very rarely coated in diseases of the pancreas. As regards the diagnosis this fact possesses interest. The secreting power of the stomach, as will be seen, undergoes a profound change in a certain direction. Enormous quantities are often secreted, in these cases, of a watery, tasteless, or sourish, thin, and ropy fluid; yet the tongue is very seldom coated. In diseases of the stomach the secretion of this organ is also changed; but the tongue is very seldom found in a perfectly natural condition." (p. 81.)

*Vomiting.* On the important symptom of vomiting we shall give the author's own observations, at length:

"With a view to a minute investigation of the vomiting which occurs in diseases of the pancreas, we have selected 39 cases, in which the morbid action had either exclusively confined itself to the pancreas, or in which there were sufficient grounds, in the slight affection of other organs at the first appearance of this symptom, for not attributing to them any important share in its production. The slightest form of this symptom was shown in those cases in which no peculiar vomiting of the food occurred, but in which a morbid excitability of the stomach and an inclination to vomit was exhibited, either by continued urgings and retchings, or by the fact that medicines having naturally no action of that kind excited vomiting. In one case, which occurred in a girl of thirteen, who died from disease of the lungs, and in whom the pancreas was found to have undergone what appeared to be tubercular degeneration, there had existed, in addition to the only symptom of disordered digestion—entire loss of appetite, this peculiarity, that the food, as soon as swallowed, was returned again into the mouth, without being rejected, by a sort of ruminating process. In all the other 35 cases there occurred more or less complete vomiting, by which food and drinks, after being taken into the stomach, were sooner or later again rejected. This symptom did not always make its appearance at the same stage of the disorder. We have before had occasion to remark that other consensual disturbances of the functions of the stomach exhibit themselves long before vomiting takes its place as one of the features of the disease. There are, however, cases in which the sympathetic excitement of the stomach betrays itself by no other symptom; indeed, in which vomiting is the only observable sign of disease during their whole progress. The *acute* forms of pancreatic disease exhibit, moreover, from their commencement violent and suddenly-occurring vomiting. To this class may be referred those cases in which, though the progress is not rapid, the development of the morbid process is of a sudden and forcible character; as, for instance, in a case narrated by Storck, under the title *hemorrhagia interna pancreatis*. In the great majority of cases this symptom comes on at a later stage of the disorder.

"The form in which this phenomenon continues to exhibit itself is not always the same. In almost all acute cases the vomiting was extraordinarily pertinacious and incessant, excited afresh by every kind of food or drink of even the mildest kind, and continuing, with the same degree of violence, up to the period of death. In one of these cases this symptom exhibited itself in a very peculiar manner. Whilst the accompanying fever retained a continued character, the vomiting was excited or moderated at irregular periods; but when, on the ninth day of the disease, the fever took a tertian intermittent form, the vomiting occurred with greater violence during the fits, and was somewhat moderated during the intermissions. After the gradual disappearance, however, of this intermitting fever, the vomiting continued in an aggravated form for two months unceasingly, and was only quieted eight days before the death of the exhausted patient, who had been kept alive during this long period by means of nutritious clysters......

"Except in one case—apparently a scirrhous degeneration of the pancreas, of an indolent character, in which the vomiting, after having lasted a long time, again disappeared—all the observations showed that this symptom, having once appeared, continued in the same or an aggravated form, seldom with a lessened degree of violence, and never entirely disappeared......

"Both as regards its frequency, and the periods at which the vomiting takes place, there is considerable variation. It seldom occurs at irregular intervals, frequently as often as twenty times a day, in cases, as it would seem, in which a copious secretion takes place into the stomach. It is more frequent in the morning on a fasting stomach, at which time, on first waking, large quantities of fluid are ejected, that had collected during the night. It occurs most frequently, however, at a fixed period after meals, from two to three hours.

"The vomiting has an effect on the other symptoms and on the general state; local pain is relieved by it, and exacerbated when it is checked. It comes on late, when digestion is much damaged, and food irritating the stomach is rejected: in this state it has the effect of diarrhea, which sometimes replaces it. Where the vomiting is violent the fluid is tinged with bile; sometimes blood is rejected,—in one case so abundantly as to cause speedy death." (pp. 81-5.)

The author remarks, that "an examination of the comparative frequency with which the other symptoms accompany vomiting, may give us an insight into the manner in which it is brought on in diseases of the pancreas." In the 39 cases selected, vomiting was accompanied by pain in 35, watery evacuations from the mouth in 17, costiveness in 13, thirst in 11, loss of appetite in 11, diarrhea in 6, it was the only symptom in 3. Notwithstanding the frequency of this symptom, the opinion maintained by many authors, that vomiting is an unfailing symptom of diseased pancreas, is erroneous. In the cases examined by the author it was found to be much more often absent than present. Indeed, as he observes, it is a symptom of an advanced stage of the disease, and when it has assumed an active state.

*Constipation.* This was present in 32 cases, and is considered by our author to be also a symptom of the inflammatory stage of pancreatic disease.

*Diarrhea* was found in 12 cases, and most generally in diseases of a malignant kind.

*Other secretions,* as of the kidneys and skin, exhibited nothing peculiar.

*Vascular excitement* was often absent even when other symptoms were very aggravated.

*Reflected affection of the nervous system.*

"It is probably to the deep situation and to the parenchymatous structure of the organ, that we should attribute the restlessness and distress as well as the other nervous affections which are met with in almost all the acute cases. It was without doubt anxiety and restlessness which drove one patient from his bed, notwithstanding his weakness, or an internal feeling of disease proceeding from the affected part which continually agitated and forced groans from another, in others caused affection of the head with slight delirium, or great faintness, or continued sleeplessness, and absence of mind."

In chronic cases sleeplessness is a very frequent symptom.

*Effect on the nutrition.* The opinion maintained by Pemberton, that pancreatic disease gives rise to a peculiar degree of wasting is confirmed, Dr. Claessen thinks, by a comparison of numerous cases. At the same time it must be admitted that several cases are recorded by Abercrombie and others, in which the body was found after death to contain rather an unusual amount of fat.

The third section of the work is devoted to the *complications* of the diseases of the pancreas.

1. *Complications with diseases of the stomach.* We have seen that the symptoms of pancreatic diseases are chiefly such as arise from sympathetic affection of the stomach. We also find after death in a certain number of cases, that the stomach has undergone alterations of structure varying in character, but generally such as are the results of inflammatory action. The question then arises, whether during life these latter cases have exhibited gastric symptoms in a more marked degree than those in which no such structural alterations are found in the stomach. To this Dr. Claessen replies as follows:

"Such an agreement between the symptoms and the results of the necropsy does not necessarily exist; a certain number of cases will be found, and amongst them some of the most exquisitely marked specimens of acute inflammation of the pancreas, which exhibit that symptom which marks the highest degree of sympathetic affection of the stomach, namely vomiting, in a degree paralleled only in acute inflammation of the stomach produced by corrosive poisons, and in which, nevertheless, the pancreas alone is found to have been diseased; in other similar cases an alteration of structure of the stomach is found indeed, but can be shown to have come on at a late period of the disease. On the other hand we find that it very rarely happens that any change of character or increased severity of the symptoms accompanies the invasion of disease of the stomach; and lastly, that very considerable changes of structure of this organ occur without the slightest disorder in its functions."

The cardiac orifice of the stomach is very rarely affected by disease of the pancreas; Rahn relates one such case. The pylorus, on the other hand, was found narrowed and indurated in 15 cases; vomiting was altogether absent in 5 of these; in the other 10 it was present; in 7 of these not in such a degree as to distinguish them from others in which no narrowing of the pylorus existed. In 2 however, related by Serval, where the pylorus was quite closed, nothing but milk and cold drinks would remain down, and in a third the stomach continued to receive food for three, four, or even five days, and then the whole undigested mass was rejected together.

2. *Complication with diseases of the liver.* This complication is nearly as frequent as the preceding. It rarely occurs that the disease begins in the liver, and extends subsequently to the pancreas, or that both organs are simultaneously affected; on the other hand, communication of disease from the pancreas to the liver is very frequent, and this may take place either by actual extension of the morbid process, or as in the case of the stomach in consequence of the physiological connexion of the liver and pancreas. Obstruction of the gall-ducts is not an unfrequent cause of jaundice, which is sometimes the first symptom to give rise to suspicion of pancreatic disease; jaundice may, however, be present without any actual obstruction of the ducts. Dr. Claessen here refers to the cases related by Dr. Bright in the 'Medico-Chirurgical Transactions,' of fatty matter voided in cases of complication of disease of the pancreas and liver, and agrees with Mr. Lloyd in considering the liver as the source of this secretion.

3. The complications of affections of the pancreas with diseases of the *spleen*, *kidneys*, *mesenteric glands*, and the other parts of the alimentary canal, are much more rare; and neither the connexion between these nor the symptoms by which their presence is indicated is sufficiently clear to merit particular exposition.

4. Much importance has been attached by some authors to the pressure made on the *aorta* by the enlarged pancreas. A variety of affections of the head and chest have been attributed to this cause, but, our author thinks, without sufficient grounds. The cases where such results were obviously due to this cause are very rare.

5. *Complication with fever.* Sylvius and his followers attributed to the pancreas a chief part in the production of intermittent fevers, and Fred. Hoffman takes a similar view. In examining the grounds of such an opinion, Dr. Claessen finds sufficient evidence of a connexion between intermittent fever and certain affections of the pancreas, and quotes some cases to show that these affections may be accompanied by an intermittent as well as a continued form of fever, but concludes by expressing the opinion that this connexion is not more intimate than between these fevers and the affections of the liver or others of the abdominal viscera.

6. *Complication with nervous diseases.* Many of the older physicians, as Fernelius, Trew, and Fr. Hoffman, ascribed to pancreatic diseases great importance in the production of *hysteria*, *hypochondriasis*, *epilepsy*, and other nervous and mental affections. By far the greater number of the cases brought in support of this view, are either so imperfectly related, as to be valueless, or the affections of other more important organs were so considerable as to prevent our coming to any conclusion as to how much might be due to the pancreas.

*Diagnosis.* Diagnosis of diseases of the pancreas, Dr. Claessen truly observes, has always been considered most difficult. "They have not only been confounded with diseases of the neighbouring organs of digestion, as the stomach and liver, an error into which Annesley appears to have fallen in some of the cases seen and described by him with extreme anatomical accuracy, but have even been sometimes mistaken for those of a very different nature, as disease of the kidneys or pulmonary consumption." This difficulty in recognizing disease of the pancreas, arises from various causes, amongst which are the following :

"1st. The difficulty not to say impossibility of arriving at any certainty respecting functional disturbance of this organ either by direct or indirect methods. The observation of inquirers has not yet been turned to the examination of the pancreatic juice in diseases. Independently of the admixture with other organic fluids which it must undergo before being evacuated, no such examination could be thought of, except in cases where it was voided in large quantities either by the mouth or anus. We have before shown it to be highly probable that the so-called *sialorhœa* of the pancreas, in truth, has its origin in the secretion from the mucous coat of the stomach, and the rarer cases in which diseases of the pancreas are accompanied with purging would probably only furnish a negative result.. The absence of any peculiar colour, smell, &c., in the pancreatic fluid prevent our recognizing its resorption, when the secretion is obstructed, as we often can in the case of bile or urine. .... The fæces probably contain no traces of it, since its constituent parts appear to be altogether of a vital and not of an excrementitial character, and would therefore be absorbed along with the chyle in its passage through the intestines. Again, the part which the pancreatic juice performs in assimilation of the food, and consequently on the nourishment of the body, is not of such striking importance as that its absence from the process of assimilation would be immediately perceived, although on this point depends the importance which has been attributed to wasting as a diagnostic mark of diseases of this organ. It would appear as we have before remarked, that its secretion when checked, is replaced by that of some other part, probably the stomach, since in complete degeneration of this gland the digestion is often not much disturbed, and after its entire abstraction in animals, nutrition is but little affected.

"2d. The depth at which this organ is placed, which bids defiance to external examination.

"3d. The slight sensibility of the organ. Not only is the pancreas itself scantily supplied with nerves, but its connexion with the more sensitive peritoneum is but slight, being clothed by it in front only, and that loosely. Hence the pain caused by inflammation of this organ is but slight, and becomes severe only under particular circumstances; the situation of it is seldom sharply defined and referred to the spot occupied by the diseased viscus, but generally vague and deceptive. The peculiar organic sensibility of this viscus also appears to be but feeble, at least, we can only in this way account for the fact that scirrhus and cancer, which in other structures, as the mammæ and testicles, cause so much pain, remains generally indolent in the pancreas. We can adduce examples of the complete destruction (verjauchung,) of this organ by cancer, in which scarcely any morbid sensitiveness existed.

"4th. Its being surrounded with organs of greater sensibility, and of more importance in the organism. The symptoms which occur in diseases of the pancreas depend partly on the sympathetic affection of other organs connected with this part, especially of the stomach; partly on this, that the morbid processes which slumbered unperceived, whilst confined to the pancreas, have now extended to neighbouring parts, and begin to excite disturbances in the system. Thus, jaundice has often afforded the first evidence of the presence of scirrhus in the pancreas. As this organ does not hold an important place in the general system, its diseases comparatively seldom end fatally without other more important parts being affected. And hence, whilst on the one hand, the group of symptoms occurring in pancreatic disease exhibits an admixture of some unconnected with this organ on the other, the number of cases of pure pancreatic disease observed is limited.

"5th. The slight influence of the diseases on the general systems of the organism, the circulation, nervous system, and secretions.

"6th. The small number of well observed and fully reported cases which we as yet possess."

The author shows that no pathognomonic symptoms exist. Vomiting was supposed by Casper to be a constant symptom of scirrhus of the pancreas, but erroneously; other authors have attached equal importance to the salivary discharge; this, however, is frequently observed. There is, in fact, no symptom which can be accounted pathognomonic; certainly none which can lay claim to be considered essential. Failing this, it has been attempted to form a diagnosis by the grouping together of a certain number of symptoms. Rahn enumerates as "diagnostic, essential, little varying, and very generally present," the following symptoms:

"A pain seated between the pit of the stomach and navel; a perceptible, hard, moveable swelling, giving rise, in the upright posture, to the sensation of a heavy body in the same spot; a constant sour, burning heartburn, extending over the whole gullet, with a copious flow of a watery, tasteless, or sour saliva; a hard dry fecal evacuation; diminished appetite and loathing for food; vomiting coming on in the course of the disease, occurring at intervals, and attended with the rejection of all the food taken, together with a slimy fluid; lastly, wasting and hectic fever, with its accompaniments.

"Pemberton observes that all the patients had complained of a greater or less deep-seated pain in the precordial region, and had felt more or less ill and had wasted; he thinks, therefore, that these appearances, where other symptoms of disease of the stomach, of the back part of the liver, of the gall-bladder, or ducts, or of the small intestines, are wanting, would denote pancreatic disease.

Kreysig says: induration of the pancreas produces the greatest disturbance of digestion; on making pressure over the situation of the stomach, a resistance will be perceived, on careful examination, such as might be caused by a narrow board; the patients have also, though not always, an increased flow of saliva in the mouth, they complain of acidity, &c. Hohnbaum considers, as the most certain signs, eructation of watery fluid; vomiting of water, especially when accompanied with vomiting of the food several hours after a meal; great thirst with a moist tongue; remarkable wasting in the absence of colliquative symptoms."

The author passes in review various other symptoms on which a diagnosis has been attempted to be founded; we shall, however, content ourselves with extracting the following summary of the differences between diseases of the stomach and those of the pancreas:

| "*Diseases of the Stomach.* | *Diseases of the Pancreas.* |
|---|---|
| The pain is readily increased by pressure; and this in proportion to the severity of the pain. | The pain is less frequently and readily increased by pressure; even when very intense, pressure generally fails to aggravate it, or even serves to lessen it. A particular position of the body on one side or the other on the back or belly, or bending forwards, &c. has an effect on the pain. |
| During this, a notable discharge of saliva occasionally takes place, with swelling and pain of the salivary glands. | The flow of water from the mouth is sometimes so copious as to have the appearance of salivation; but this is never attended by swelling of the salivary glands. |
| The ravenous appetite found in some cases is often accompanied by faintings, pains of the head, oppressed breathing, which continue until it is appeased. | Increased appetite is sometimes seen, but never attended with such sympathetic affections. |
| The tongue, besides being coated in various ways, is not unfrequently dry and red; the mucous membrane of the mouth is also often beset by aphthæ. | The tongue is generally clean, or at most, and that rarely, has a thin white or yellowish coating. |
| Bloody, chocolate-coloured, or coffee-ground fluids are often vomited with little effort, and in diseases of very chronic character. | Vomiting of such fluids is extremely rare, and occurs only in acute cases or with violent retching. |
| In affections of the cardia, the food is rejected immediately after it has been swallowed. When the pylorus is narrowed, the vomiting has generally a mechanical character, without being attended by other stomach symptoms. Vomiting is often the only symptom. | The vomiting is almost always attended by other disturbances of the functions of the stomach, and has never a simply mechanical character. |
| Evacuations, caused by administering purgatives, generally aggravate the disease. | They relieve the disorder. |
| The secretion of the skin is frequently altered, generally lessened, or suppressed, or eruptions take place on it. The urinary secretion is very variously modified. | The secretions of the skin and kidneys are very rarely altered, and then only in consequence of disturbance of the circulation. |
| Digestion is sometimes attended with feverish excitement, cough, and dyspnea, or sympathetic palpitations of the heart, are present, and these are aggravated or lessened with the stomach affection. | Both kinds of symptoms are entirely wanting; on the other hand, in a few cases, pulsation is seen or felt in the epigastrium, or a murmur is heard there, isochronous with the heart's stroke. |
| Digestion is frequently attended with a sense of weariness, oppression, and pain in the limbs, &c., or other nervous affections are found, as pain in the head, hypochondriasis, &c. | Such symptoms are never found *during digestion;* sometimes during the course of the disease aberration of mind has occurred. |
| The wasting is not great, it exists in proportion to the duration of the disease. | The highest amount of wasting is not unfrequently seen; sometimes it comes on suddenly. |

During their progress, they show great diminutions of intensity, sometimes without evident cause, and sometimes under the influence of internal or external causes. Their progress is more rapid. Crises are more frequently seen.

They are more within the reach of medical treatment, and are affected by such agents.

When they have reached a certain stage, they assume a more stationary condition, with a less amount of susceptibility to external and internal influences. Their duration is very prolonged, and they show no signs of crisis.

To judge from facts hitherto known, it is more difficult to affect their progress by therapeutic agents."

*Prognosis.* This must be unfavorable, if we are to judge from what we as yet know of diseases of the pancreas; at the same time, although the termination may be unfavorable, the progress is generally slow, sometimes extraordinarily so. The prognosis will also depend upon the stage of the disease, the severity of the symptoms, and the nature of the disease whether malignant or not; and also on the diseases of other parts with which it may be complicated.

*Treatment.* This, at present, must be founded on general principles, as experiment has not as yet discovered any means specially fitted for overcoming disease in the pancreas. The subduing inflammation by leeches, bleeding, &c.; the use of mercury to overcome chronic inflammation, &c.; the removal of congestion, &c. by the use of saline mineral waters, &c.; probably iodine and its compounds—such are the weapons with which Dr. Claessen proposes to cope with these diseases.

In the Second Part of his treatise, Dr. Claessen proceeds, in the same systematic and minute manner, to treat of the special diseases of the pancreas. Our space will not allow us to notice this portion of the work so fully as we have done the former.

1. *Simple hypertrophy* of the pancreas is so rare a disease that our industrious author has not found on record a single unequivocal case of it.

2. *Atrophy.* Several cases are narrated in which atrophy, or rather the conversion of the gland into fat, had followed the abstraction of the ordinary stimuli by closure of the pylorus, or from other diseases of the stomach or liver in which the food had been rejected. Conversion of the *left* end into fat is sometimes due to obstruction of its secretion, from the duct being pressed on by scirrhus of the *head* or *right* extremity of the gland.

In cachexia and dyscrasia, the wasting of the pancreas is very common.

In people of weak intellect (blödsinnig), in whom the vital functions, and especially the digestive, are often in a very depressed state, the pancreas has been found so wasted as to have been supposed to be altogether absent. Certain cases of wasting of the pancreas are related, accompanied by hardening of the structure, and ossification of the vessels; but Dr. Claessen considers these changes as the result of chronic inflammation and not of wasting or atrophy properly so called.

3. *Calculus.* Sometimes this change appears to take place without any other morbid alteration in the gland or elsewhere, as in cases related by Lieutaud and Baillie. Galeati mentions an instance of a man who had suffered for thirty years from pain in the precordia, vomiting, costiveness, and repeated attacks of jaundice, in whom, after death, the head of the pancreas was found changed into a sac as large as a hen's egg, containing twenty small calculi. Portal relates a case: The pancreas contained a dozen stony concretions, of which some were as large as a nut. The duct was so much enlarged as to lead to the supposition that some of the stones must have been evacuated. These concretions appear to be composed of carbonate of lime and phosphate of lime in variable proportions.

4. *Hyperemia.* Under this head an interesting case is quoted by our author from Storck, of a woman twenty-eight years of age, in whom, vomiting coming on suddenly and violently during the menstrual period, the discharge ceased. Difficult breathing, cold extremities, and palpitation followed—then faintings. After a month, pulsation at the epigastrium, with weight; then, costiveness and want of sleep. To these succeeded vomitings and bilious diarrhea—great wasting—death. The pancreas weighed thirteen pounds, and contained a sac filled with coagulated blood. Probably disease of the vessel had before existed, and rupture took place on the cessation of the menses, giving rise to a sort of aneurism in the gland.

5. *Inflammations.* Authors agree in stating that acute inflammation of the pancreas is an extremely rare disease. Andral says its existence has never yet been proved by necropsy. This, however, if strictly true, does not disprove the existence of such a disease, since, as M. Mondieù and our author observe, the pancreas is not an organ of such great importance in the economy as that inflammation of its structure would be likely to prove fatal during the acute stage.

Hitherto the history of acute pancreatitis, as given by systematic writers, has been founded on two or three cases, in only one of which, related by Schmackpfeffer, did death take place before the affection had decidedly passed into a chronic state. Even in Schmackpfeffer's case, a month had elapsed from the first appearance of symptoms of acute inflammation of the pancreas, before the patient was carried off by a pleuritic affection, and the original symptoms had, in the mean time, considerably subsided; nevertheless, the state of the organ after death was such as to leave no doubt of the nature of the attack. In this instance, the pancreatic disease supervened on an inflammatory state of the mucous membrane of the stomach and bowels, the consequence of salivation by mercury for the cure of syphilis. Another case, bearing considerable resemblance to Schmackpfeffer's, as regards the symptoms, is related by Juppin in the 'Journal de Médecine,' but here a still longer period elapsed before the disease proved fatal. Both the above cases possess much interest from the well-marked character of the symptoms, and are given at length by Dr. Claessen; they are, however, rather too long for quotation, and we shall therefore select his third case, extracted from Casper's 'Wochenshcrift,' as offering a well-marked instance of acute inflammation supervening on chronic pancreatitis.

"Case. A Frenchman, of athletic build, forty-five years of age, of temperate habits and happy temperament, having always enjoyed good health, was suddenly seized with severe pain in the belly which became much distended, and with violent vomiting of a green bilious fluid, renewed as often as he swallowed any liquid, even water. His ordinary medical attendant bled him: the blood appeared healthy. During the night Dr. Casper was also called to see him. The patient was at this time suffering from obstinate constipation, notwithstanding the administration of various purgatives. His belly was greatly distended, and he complained of constant severe pain and tenderness a hand's breadth above the navel. Elsewhere the belly was scarcely tender, and no hardness could be perceived, the patient being very fat. Everything swallowed was immediately rejected with a full stream of greenish fluid. With a moist though foul tongue he had constant thirst, which he, however, dreaded to gratify. He tossed restlessly from one side to the other, crying out constantly, " j'étouffe, j'étouffe!" an expression denoting rather great discomfort and distress than a want of breath. His pulse was eighty-five, neither full and hard nor, on the other hand, small and oppressed.

"The *diagnosis* was obscure. His first attendant leaned to the opinion, that inflammation existed in the abdominal viscera; but the state of the pulse, and the entire absence of inflammatory appearances in any of the blood drawn at three several times, as well as the absence of any beneficial effect from this and other antiphlogistic means, led Dr. Casper to doubt the correctness of this view. The disease continued its course, unaffected by treatment, for four days, at the end of which time the patient died delirious.

"*Dissection.* Neither the peritoneum nor intestines were inflamed. The liver was free from disease, nor were any gall-stones found. All other organs were healthy, except the pancreas, which was swollen to the size of a man's fist, of cartilaginous hardness, strongly adherent to the stomach and duodenum, and of a brownish red hue. The internal structure was no longer perceptible."

The suddenness and violence of the attack—the deep-seated pain—the vomiting—the urgent thirst—and the extreme restlessness justified the opinion that inflammation was present in the abdomen; whilst the seat of the pain, "a hand's breadth above the navel,"—the moist tongue—the absence of reaction in the circulation, as shown by the state of the pulse and the blood, peculiarities also present in Juppin's case—might serve to distinguish the affection from acute gastritis.

Dr. Claessen contrasts the symptoms of acute pancreatitis and gastritis as follows:

| "*Acute Pancreatitis.* | *Acute Gastritis.* |
|---|---|
| The pain is situated deep behind the stomach in the flank (weichen), it may be violent, but is dull, heavy; it gives rise to a peculiar distress and restlessness, or is attended by great faintness. It holds no proportion to the other symptoms, such as vomiting. Pressure does not increase it in any remarkable degree. | The situation of the pain is more superficial; it is tearing, cutting; it is the most urgent symptom. Pressure increases it. |
| A swelling is not perceptible even when the pancreas has undergone considerable enlargement, on account, as it would appear, of the accompanying distension of the belly, even when this is inconsiderable. | The distension and upward pressure of the belly are more considerable. |
| The vomiting is especially violent; in proportion to its severity, greater or less quantities of green fluid are evacuated. | Probably only in cases of inflammation from corrosive poisons does the vomiting attain such a degree of violence; but little bile is evacuated. |
| Thirst and constipation are common to both disorders, and in similar degree. | |
| The tongue always moist, either clean or has a light white coating. | The tongue is dry and red, or coated with thick dry crusts, or aphthæ are seen all over the inside of the mouth. |
| The appetite is little affected, it may be natural or even greater than usual. | The appetite fails altogether, and disgust is exhibited at all kinds of food. |
| The vascular system takes little part in the disease; the attendant fever is very slight. | In the vascular system a reaction shows itself, answering to the violence of the inflammation; and other feverish symptoms are present. |
| Strong purgatives and even emetics do not aggravate the symptoms; the latter even seem to have a temporarily beneficial effect. | Emetics increase the pain; only such purgatives are borne as do not irritate the inflamed surface." |

No instance is on record of *abscess* in the pancreas as a result of acute inflammation nor of gangrene.

In treating of *chronic inflammation* of the pancreas, Dr. Claessen insists on the necessity for distinguishing those cases in which the organ has simply acquired increased hardness without undergoing any other change from those in which hardness is the result of chronic inflammation, and is attended with the other alterations usually produced by inflammation.

He considers that the pancreas, like the salivary glands, may exhibit various degrees of size and hardness, within the limits of its normal state, and consequently without giving rise to any symptoms during life. The induration produced by chronic inflammation requires also to be distinguished from that produced by scirrhus, and our author has at some length pointed out what he considers to be the distinguishing marks between these two forms of disease as exhibited in the pancreas. We believe, however, that it will be found more easy to draw up such formulæ on paper, than to decide, with the disease before us and the scalpel in hand, exactly under which head an individual case is to be placed.

Nine cases of chronic pancreatitis, more or less fully reported, and possessing various interest, are given by Dr. Claessen from various sources, and serve well to illustrate this form of disease. We give the summary of the symptoms observed in these cases, in the author's own words:

"All the specimens of this class agree entirely in their symptoms, not only with each other, but with the symptoms before described as marking a case of acute inflammation terminating in a chronic state. They differ from it only in the first stage of the development of the disease as well as slightly from each other. Whilst in all, the progress was at first slow and insidious; in one local pain was for years the only symptom; in another there was weak digestion, requiring a careful attention to diet, followed soon by heartburn; in a third periodical pains in the stomach; and, lastly, in another complete vomiting, by which even from the first large quantities of fluid were evacuated, was the earliest symptom. When the form of the disease had become more fully developed by the appearances of other symptoms, we find that in none of the cases related was any one of its peculiar characters absent; on the contrary, it exhibits throughout a remarkable uniformity in the number and nature of the symptoms. In the first place there was *pain*, either in the epigastrium, or in some situation pointing more especially to the diseased organ, increased by external impressions, as pressure, a full state of the stomach or colon, the position of the body; and, secondly, there was *copious evacuations from the mouth of watery and slimy fluids*, which in its passage through the œsophagus, was sometimes preceded by burning pain at the pit of the stomach, and at a later period subsequently attended by actual *vomiting*. In others, no pain existed, but the passage of the fluid from the stomach was evident to the patient; or lastly in others, though from time to time the evacuation of the fluid was attended with retching, it frequently gathered so insensibly in the mouth, as to mislead the patient and the medical observer as to its source. Thirdly, in most of the cases, *there was diminution or entire failure of the appetite*. Fourthly, there was in all, without exception, the most *obstinate costiveness*, sometimes giving way for a few days, or towards the end of the disease, being supplanted by a diarrhea. Fifthly, there was generally *considerable thirst* with a moist and clean tongue, or with a tongue having a yellow coat. Sixthly, in all there was *wasting*, generally to a great degree, together *with but slight affection of the vascular system, the secretions, or the nervous system*."

When the pancreatic disease is complicated as it frequently is, with disease of the stomach or of the liver, the difficulty of arriving at an accurate diagnosis will necessarily be much increased.

*Suppuration* in the pancreas has been described by many authors, and there seems no doubt that chronic inflammation of the pancreas may terminate in this manner. The symptoms to which it gives rise are, however, unknown; if there be any beyond those attendant on chronic inflammation generally.

A few cases are on record of *gangrene* of the pancreas, the result of chronic inflammation. One of these is related by Portal in his 'Anat. Médicale,' which appears to have been a case of chronic disease attacked by acute inflammation terminating in gangrene after twenty days. The symptoms of this also are too obscure to furnish grounds for diagnosis.

*Anormal hardness* of the pancreas we have before stated is to be distinguished from chronic inflammation. Our author has found forty-eight cases on record, in which the pancreas had acquired this indurated state. In most of these no symptom had led to a suspicion of the existence of disease of the pancreas. Scholler met with an instance of this kind in an infant only nineteen days old, so that there can be no doubt this state of the pancreas existed during fetal life.

*Morbid softness* of the pancreas has been occasionally met with but rather as dependent on some general disease of the body, as scurvy, scrofula, &c., than as an original affection of this organ.

*Pseudo-morphous diseases.* The following diseases of this class have been found in the pancreas: Scirrhus, medullary tumour, steatoma, tubercle, melanosis, hydatids. The symptoms caused by these several diseases are very similar, and may be arranged under two stages. In the first, the symptoms are few, and are rather of a mechanical kind arising from the weight of the tumour, or its pressure on other organs, as the stomach or liver, and giving rise to vomiting, to jaundice, &c. In the second stage when an inflammatory action has come on in the part, symptoms arise such as have been described under the head of chronic inflammation.

*Scirrhus.* After making all allowances for the erroneous descriptions of some of the older authors by which various diseases were classed under the term scirrhus, this still appears to be the form of disease most frequently met with in the pancreas. The symptoms exhibited by scirrhus of the pancreas vary greatly in number and intensity in different cases. This difference Dr. Claessen considers to be owing to the stage at which the disease has arrived, and to the presence or absence of inflammatory action. "As long as inflammation is not present in the morbid structure, nor is excited in the surrounding parenchyma of the organ by the presence of this parasitic formation, we have merely an indolent swelling, which has no other effect than to put a stop to the function of the organ. Hence it has not unfrequently occurred that a scirrhus of the pancreas has been unexpectedly found in the bodies of persons dying of the most varied diseases." (pp. 278-9.) The symptoms, when any are present, are rather of a mechanical kind, arising from the weight or pressure of the tumour on neighbouring parts. "As soon as the inflammatory process establishes itself in the tumour or in the neighbouring parts, the various anormal conditions of sensation and sympathetic affections of the stomach come on, by which the disease obtains, more or less, the character of chronic inflammation of the pancreas."

Dr. Claessen has selected 16 cases of scirrhous pancreas from various sources, to illustrate the disease in its two stages, or as complicated with inflammation to a greater or less degree; and has endeavoured from these to elicit the means of diagnosis between scirrhous and chronic inflammation. We fear, notwithstanding these endeavours, we must be contented, in many cases, still to remain in doubt respecting the nature of the disease, since the difference is, after all, rather in the degree in which the same symptoms are present in the two diseases than in the difference of the symptoms. We shall, however, give the parallel with which our author concludes his examination :

| "*Chronic Pancreatitis.* | *Scirrhus of the Pancreas.* |
|---|---|
| The form of the disease (krankheitsbild) comprises very constantly all the symptoms* united. | The group is never complete. When the other symptoms are found, vomiting is generally wanting, and constipation. |
| A swelling is less frequently to be distinguished; it comes on in a late stage of the disease, is of a smaller size, and is not in proportion to the severity of the symptoms. | A swelling is more frequently found, and is of a larger size; often it is the first sign of the disease noticeable; it holds more proportion with the other symptoms. |
| A single symptom is never found unaccompanied by others. | Often only one symptom is present, and that is most frequently *pain.* |
| The pain generally precedes swelling, but is preceded by other sympathetic stomach symptoms. | The pain is often periodical, and appears in paroxysms. Often it is relieved by pressure, or by taking food. |
| These sympathetic symptoms bear a pretty close relation to the severity of the pain. | The pain exceeds all the other symptoms, often in a remarkable degree. |
| They either make their invasion at the same time as the pain, or precede it. | When sympathetic stomach symptoms occur, it is generally long after the pain. |
| Vomiting of the food is very constantly present. | Vomiting of the food alone is very seldom found. |
| The watery evacuations from the mouth are moderate and tasteless. | The fluid rejected from the mouth is sometimes profuse or remarkably sour. |
| The appetite is always lessened, and thirst generally increased. | The appetite is often normal, sometimes decidedly increased; which gives a contradictory character to the symptoms. Thirst is rarely present. |
| The constipation is urgent and enduring. Diarrhea is found only at the commencement or termination of the disease. | The constipation is transitory: it generally alternates with diarrhea; sometimes the latter alone is found, and has a colliquative character. |
| Wasting is always present, in a greater or less degree; but follows the other symptoms slowly, gradually, and towards the close of the disease, and is in proportion to their severity. | Wasting is sometimes entirely wanting; even an undue amount of fat may be found. But often the wasting bears no proportion to the small amount of suffering, or comes on very early." |

We must observe, however, that some of these statements appear to be hardly borne out by the very cases which Dr. Claessen has quoted; and we may add, that though occasionally the aspect of the patient or the prominence of some peculiar symptom—as the vomiting of a peculiarly acrid fluid, colliquative diarrhea, &c.—may make us pretty certain of the existence of malignant disease, we feel assured that our means of diagnosis are by no means sufficiently advanced to enable us, in the great majority of cases, to determine whether the case is one of chronic inflammation, or scirrhus, even should we have overcome the previous difficulty of determining what organ is really affected.

* Those namely which have been found to be so often present as to deserve to be considered as essential, namely, pain, vomiting, watery evacuations from the mouth, constipation, thirst, loss of appetite, and wasting.

